# Non integrative strategy decreases chromosome instability and improves endogenous pluripotency genes reactivation in porcine induced pluripotent-like stem cells

**DOI:** 10.1038/srep27059

**Published:** 2016-06-01

**Authors:** Annabelle Congras, Harmonie Barasc, Kamila Canale-Tabet, Florence Plisson-Petit, Chantal Delcros, Olivier Feraud, Noufissa Oudrhiri, Eva Hadadi, Franck Griscelli, Annelise Bennaceur-Griscelli, Ali Turhan, Marielle Afanassieff, Stéphane Ferchaud, Alain Pinton, Martine Yerle-Bouissou, Hervé Acloque

**Affiliations:** 1 INRA, UMR 1388 Génétique, Physiologie et Systèmes d’Elevage, F-31326 Castanet-Tolosan, France; 2 Université de Toulouse INPT ENVT, UMR 1388 Génétique, Physiologie et Systèmes d’Elevage, F-31076 Toulouse, France; 3 Inserm, UMRS935 ESTeam Malignant and Therapeutic Stem Cell Models, F-94800 Villejuif, France; 4 Service d’hématologie, APHP, GHU Paris Sud, France; 5 Faculté de Pharmacie de Paris, Université Paris Descartes, Sorbonne Paris Cité, Paris, France; 6 Département de biologie et de pathologies médicales, Institut Gustave-Roussy, Villejuif, France; 7 Univ. P. Sud, Univ. Paris Saclay, UFR de Médecine Kremlin Bicêtre, France; 8 Inserm, U846 Stem Cells and Brain Research Institute, F-69675 Bron, France; 9 INRA, UE 1372 GenESI Génétique, Expérimentation et Système Innovants, Surgères, France

## Abstract

The pig is an emerging animal model, complementary to rodents for basic research and for biomedical and agronomical purposes. However despite the progress made on mouse and rat models to produce genuine pluripotent cells, it remains impossible to produce porcine pluripotent cell lines with germline transmission. Reprogramming of pig somatic cells using conventional integrative strategies remains also unsatisfactory. In the present study, we compared the outcome of both integrative and non-integrative reprogramming strategies on pluripotency and chromosome stability during pig somatic cell reprogramming. The porcine cell lines produced with integrative strategies express several pluripotency genes but they do not silence the integrated exogenes and present a high genomic instability upon passaging. In contrast, pig induced pluripotent-like stem cells produced with non-integrative reprogramming system (NI-iPSLCs) exhibit a normal karyotype after more than 12 months in culture and reactivate endogenous pluripotency markers. Despite the persistent expression of exogenous OCT4 and MYC, these cells can differentiate into derivatives expressing markers of the three embryonic germ layers and we propose that these NI-iPSLCs can be used as a model to bring new insights into the molecular factors controlling and maintaining pluripotency in the pig and other non-rodent mammalians.

Derivation of porcine pluripotent cells is of huge interest for producing transgenic animals, for modeling embryonic development as well as human and pig pathologies. The successful development of induced pluripotent stem cells (iPSCs) in both mouse and human^1^,^2^ was followed in last years by a huge effort to produce iPSCs from livestock animals for which it represents a good alternative to embryonic stem cells (ESCs) derivation^3^. Establishment of proper porcine ESCs has proven to be particularly difficult for many reasons including differences in early embryonic development and poor definition of culture medium (for review see^4^,^5^,^6^). Those experiments raised several questions about the state of porcine development in which pluripotent stem cells (PSCs) can be observed, the way to maintain this pluripotency *in vitro*, and the biological pathways upon which pluripotency depends.

The first porcine iPSCs (piPSCs) lines have been produced by retroviral or lentiviral overexpression of the four Yamanaka’s factors (Oct4, Sox2, Klf4 and Myc)^7^,^8^,^9^. These cell lines were able to form teratoma but no chimeras and the reprogramming factors were not properly silenced. Since then, several studies have focused on the derivation of porcine iPSCs by several means including plasmids, transposons or episomal vectors to counteract the effects of viral integration^10^,^11^,^12^,^13^. Nevertheless, silencing of exogenous reprogramming factors was rarely reported excepting in dox-inductible systems^8^. If almost all piPSC lines produced were able to form teratomas, only a few were described to contribute to chimeras development and to date without germline transmission^14^. This restricted pluripotency, also observed in other species, may be due to inadequate culture condition or an incomplete reprogramming that could result in part of continuous transgene expression.

As the dynamics of pluripotency during the pig embryonic development seems to be different from the one observed in mouse with both common and specific effectors and pathways^15^,^16^ the development of authentic iPSCs from different cell type and phenotype is still a challenge in the pig. Here we report the derivation of iPS-like cells from an azoospermic boar carrying a reciprocal translocation t(Y; 14)^17^. These cell lines are of great interest to assess the reprogramming potential of abnormal cell lines but also to develop *in vitro* model to study large animal cell differentiation and physiology as well as to study the effects of chromosome rearrangements in pathologies like infertility due to t(Y; 14) translocation. By using two different reprogramming techniques, the first one leading to the integration of exogenous gene in the host genome and the second one being non-integrative, we were able to generate different iPS-like cell lines (I-iPSLCs and NI-iPSLCs) harboring different profile of pluripotency. These results enabled us to investigate the effects of the reprogramming technique on genomic stability and differentiation of porcine reprogrammed cell lines and to identify the most adapted protocol for the production of a library of piPSCs with different phenotypic and karyotypic profiles.

## Results

### Derivation of putative porcine iPS cell lines from normal and t(Y; 14) fibroblasts using retroviral and lentiviral vectors

Testicular fibroblasts from an infertile boar carrying the t(Y; 14)^17^ reciprocal translocation were infected with the lentiviral construct EOS, which was used as a pluripotency reporter^18^. Overexpression of the four human reprogramming factors – hOCT4, hSOX2, hKLF4 and hMYC – was then conducted by retroviral infection. iPS-like colonies appeared after 10 days post-infection in the case of t(Y; 14) fibroblasts, were picked after puromycin selection for three days and subsequently cultivated on STO feeder cells in bFGF medium. Sixteen piPS-like cell clones were obtained, of which 14 expressed both the GFP (EOS) and alkaline phosphatase (AP). All subsequent studies were performed on piPS cell lines named I3 and I4. In parallel we produced another cell line (I20) derived from amniocytes of a fertile sow with normal karyotype and reprogrammed using lentiviral vectors coding for the six human reprogramming factors (hOCT4, hSOX2, hKLF4, hMYC, hNANOG and hLIN28).

### Morphological and molecular characterization of I3, I4 and I20 cell lines

**Figure 1:**
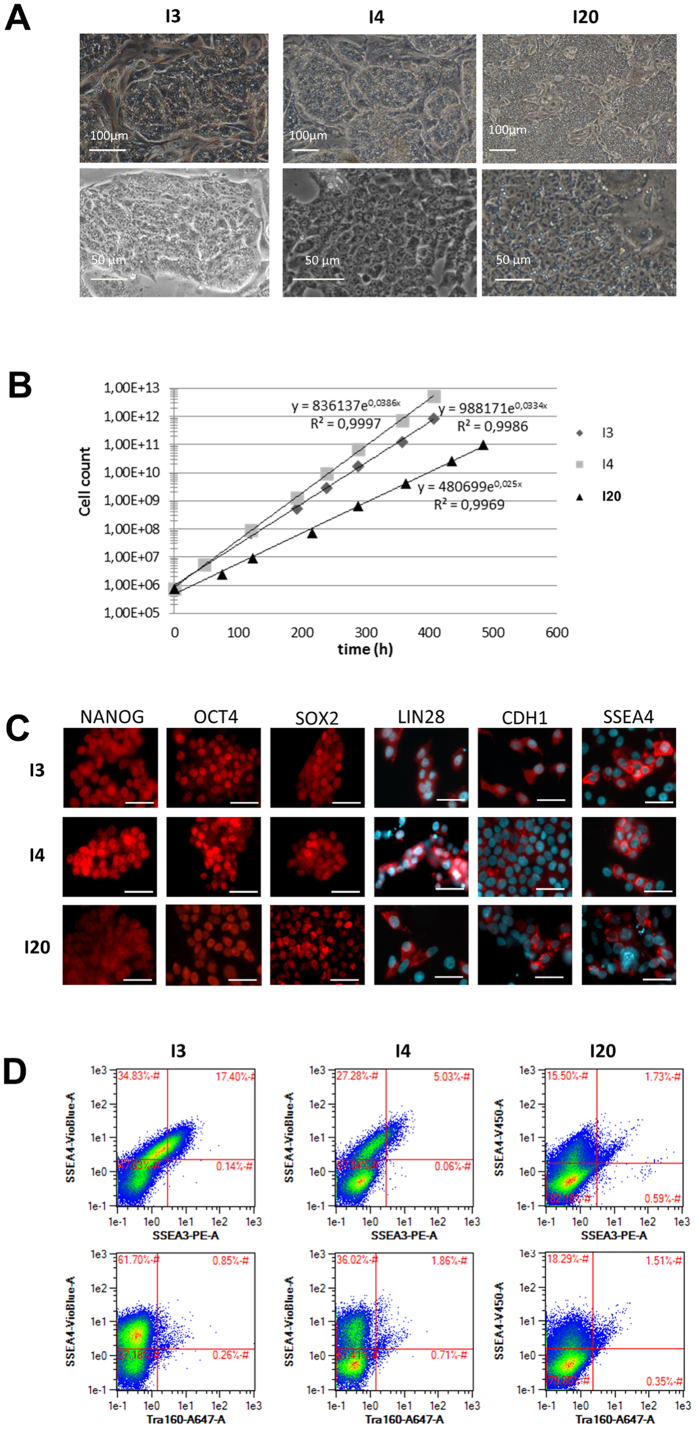
Porcine cell reprogramming using an integrative technique. (**A**) Phase contrast image of I3, I4 and I20 lines harboring an iPS-like morphology on feeder cells. (**B**) Proliferation curve of I3, I4 and I20 lines on feeder cells. (**C**) Immunoflurorescence analysis revealing the nuclear expression of NANOG, OCT4 and SOX2 (Alexa Fluor (AF) 594) and the extra-nuclear expression of LIN28, CDH1 and SSEA4 (AF594 and DAPI nuclei counterstaining), scale bar: 25 μm. (**D**) Flow cytometry immunofluorescence analysis indicates the expression of pluripotency-related surface antigen SSEA4 in a subpopulation of cells (around 55% in I3, 35% in I4, 18% in I20) while SSEA3 and TRA160 are less represented.

The three cell lines exhibit a typical morphology that resembles the one of human PSCs: they form dense colonies composed of small and tightly packed cells with a high nucleus/cytoplasm ratio (Fig. 1A). The doubling time of the populations ranged from 17 to 26 hours depending on cell line (Fig. 1B). Immunocytochemistry revealed the expression of NANOG, OCT4, SOX2, LIN28 and CDH1 in virtually all cells of the 3 populations while the expression of SSEA4 was restricted to a subset of cells (Fig. 1C). This result was confirmed by flow cytometry, showing heterogeneous expression of SSEA4. SSEA3 was also found to be expressed in a small population of cells in the I3 and I4 lines (17 and 5%, respectively) while TRA-1-60 and SSEA1 were not detected (Fig. 1D and Supplementary Fig. S1A).

**Figure 2:**
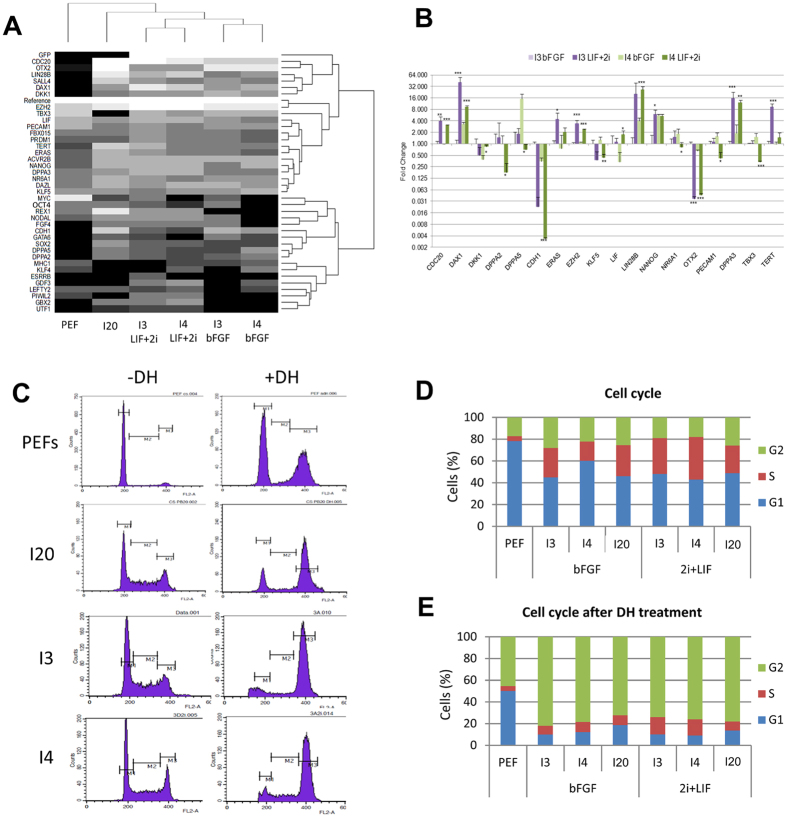
Gene expression and cell cycle properties of Integrative iPS-like cells. (**A**) I3 and I4 lines share a close expression profile of pluripotency-related genes depending on the culture media while the expression profile of I20 and non-reprogrammed cell lines are more distant. Black and white gradations highlight downregulated and upregulated genes, respectively. (**B**) Variation in expression level in a subset of pluripotency-related genes in I3 and I4 lines upon transition to the LIF+2i medium. Results are normalized on the expression in the I3 line cultured in bFGF-containing medium (log scale, t-test *p < 0.05 **p < 0.01 ***p < 0.001). (**C**) Cytograms representing the distribution of cells in each phase of the cellular cycle in non-reprogrammed and reprogrammed cell lines with or without double strand break induction (M1 = G1 phase; M2 = S phase; M3 = G2/M phase; DH = Doxorubicin hydrochloride). (**D**) Shortened G1 phase and extended S phase in reprogrammed lines cultured either in piPS or LIF+2i culture media compared to non-reprogrammed PEFs. (**E**) Absence of the G1/S cell cycle checkpoint in I lines assessed by the non-retention of cells in G1 phase after DSB induction by DH treatment compared to the PEF cell distribution.

We then studied the expression profile of 44 pluripotency genes by real-time PCR in the three putative piPS lines by using a 48 × 48 Dynamic Array IFCs (Biomark HD, Fluidigm) (Fig. 2A). This experiment revealed that the expression of the majority of the selected genes was lower in porcine embryonic fibroblasts (PEFs) than in the reprogrammed cell lines (Fig. 2A). While all reprogrammed lines express several pluripotency genes like *NANOG, SOX2, LIN28B, CDH1, SALL4, DAX1, TERT, CDC20, DAZL* and *GATA6*, I3 and I4 cell lines do not cluster together with the I20 cell line (Fig. 2A).

### Modification of the expression profile in LIF+2i condition

We then checked the ability of the 3 putative iPSC lines to adapt to a culture medium specific of naïve murine pluripotency. This medium, referred here as LIF+2i, contains leukemia inhibitory factor (LIF) to stimulate the LIF/JAK/STAT naïve-specific pluripotency pathway and is supplemented by a MEK inhibitor (PD-0325901) and a GSK3β inhibitor(1-azakenpaullone) (2i) which respectively blocks the bFGF/MEK/Erk primed-specific pluripotency pathway and activates the Wnt signaling pathway. I3, I4 and I20 cell lines quickly get used to this new culture conditions and exhibited morphological changes as soon as the first passage, with domed-like colonies and the border between cells becoming less distinct (Supplementary Fig. S1B). In I3 and I4 cell lines, the expression of the core pluripotency factors NANOG, SOX2, OCT4, LIN28, CDH1 and SSEA4 was persistent (Supplementary Fig. S1C). We then studied the expression profile of the 44 pluripotency genes by real-time PCR on a 48 × 48 Dynamic Array IFCs (Biomark HD, Fluidigm) in I3 and I4 cell lines cultured either in LIF+2i or bFGF conditions (Fig. 2A).

After more than 30 days of cell culture in LIF+2i medium, genes *UTF1, PIWIL2, REX1* and *GBX2* were found to be activated in both lines and *ESRRB* was activated specifically in the I3 cell line (Fig. 2A) while the expression of *NODAL, LEFTY2*, and *GDF3* was either activated or significantly increased (Fig. 2A). The expression of *CDC20, DAX1, EZH2* and *DPPA3* (also known as *STELLA*) also significantly increased in lines I3 and I4, like for *LIN28B, TERT* and *ERAS* even though significant differences were validated only in one cell line (Fig. 2B). Among those genes harboring an increased expression level in LIF+2i medium, several have been described as representative of the naive pluripotency, indicating a possible switch toward naïve-like pluripotency in these putative piPSCs. According to this hypothesis the primed marker *OTX2* harbored a decreased expression level in LIF+2i medium (Fig. 2B) but surprisingly the expression level of *CDH1* decreased in the LIF+2i condition (Fig. 2B).

### Cell cycle modifications

Analysis of the cell cycle revealed a shortened G1 phase in the I20 line (46%) and in the I3 and I4 lines in both culture conditions (bFGF medium: 45 and 60%, respectively; LIF+2i medium: 48 and 43%, respectively) compared to PEF (78%) (Fig. 2C,D). Upon induction of DNA double strand breaks by doxorubicin hydrochloride (DH), reprogrammed cell lines behaved differently from non-reprogrammed cell lines. For PEFs, cells were preferentially found in G1 and G2 phases in an equivalent manner (50% and 46%) while the S phase was short (4%). In I3, I4 and I20 cells were preferentially found in the G2 phase (82%, 79%, 72%) while both G1 and S phases were short (around 10% each) for the three lines, indicating a loss of the G1/S cell cycle checkpoint which controls the reparation of DNA damages. The loss of this checkpoint was also conserved in LIF+2i medium (Fig. 2C,E).

### Chromosomal instability of the integrative pig iPSLCs

**Figure 3:**
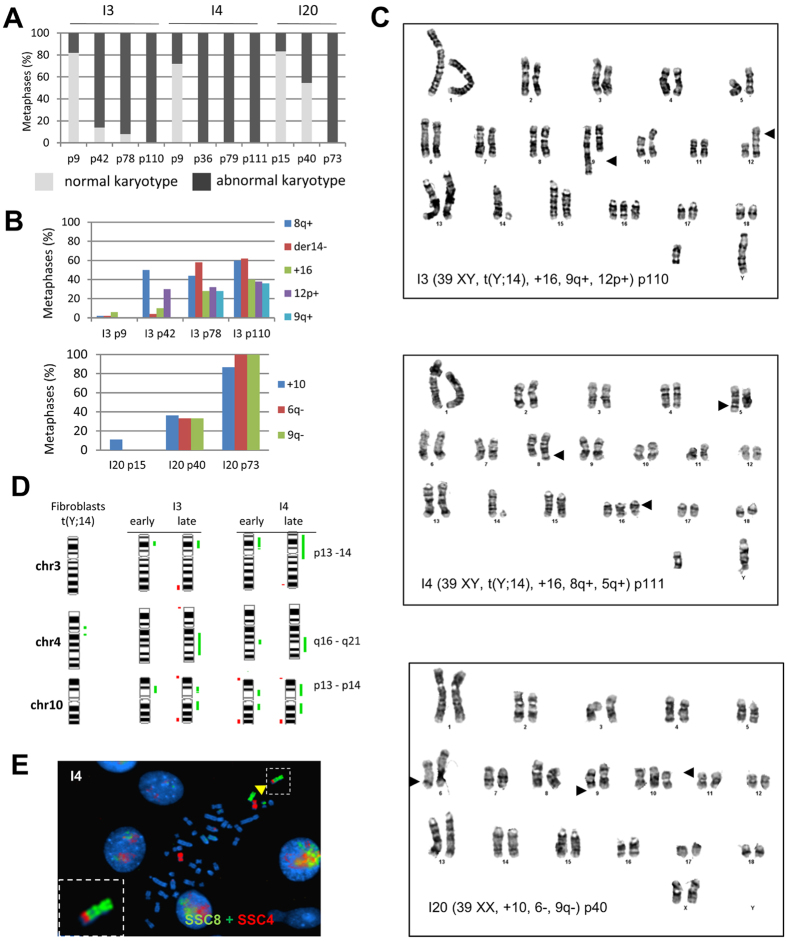
Chromosomal instability of integrative iPS-like cells. (**A**) Increase of abnormal karyotypes with passaging in I3, I4 and I20 lines. Light grey represents the percentage of normal karyotypes and dark grey the percentage of abnormal karyotypes. (**B**) Evolution of the main rearrangements observed in I3 and I20 lines. (**C**) Examples of the most represented abnormal karyotypes in I3, I4 and I20 lines at late passages. Arrows indicate the rearrangements. (**D**) Identification of common genomic duplication on chromosomes 3, 4 and 10 in both I3 and I4 lines by CGH analysis (green lines = duplications; red lines = deletions). (**E**) FISH analysis using painting probes SSC8 (green) and SSC4 (red) revealed the translocation of a duplicated fragment of chromosome 4 at the extremity of chromosome 8 with was not labeled by SSC8 probe (yellow arrow).

Karyotype of each cell line was analyzed by GTG-banding at different time points during cell culture, from early passages (<p20) to late passages (>p70). As depicted in Fig. 3A, the three cell lines reprogrammed by retroviral infection accumulated quickly chromosomal abnormalities. In early passages, it concerned around 20% of the cell population. This number increased dramatically in lines I3 and I4 in which the percentage of cells harboring chromosomal abnormalities reached more than 80% of the I3 population at passage 42 and all cells in the I4 cell line at passage 36. The three cell lines exclusively harbored abnormal karyotypes at late passages (Fig. 3A, Table 1 and Supplementary File S1). Detailed analysis of the emergent chromosomal rearrangements realized on 50 metaphases at each passage and in each cell line revealed that some rearrangements were found in a percentage of metaphases increasing over time (Fig. 3B, Supplementary File S1). These abnormalities were mainly trisomies and small addition or deletion on one of the two homologous chromosomes that were noted by chromosome number, chromosome arm (p, q) and + or − sign to indicate addition or deletion of chromosomal material. This fine analysis leads to the identification of the main karyotypic profiles in the I3, I4 and I20 lines (Table 1 and Supplementary File S1). The most represented in the I3 line were the (37XY, t(Y; 14), der14−, 8q+) karyotype concerning 38% of the metaphases and the (39XY, t(Y; 14), +16, 9q+, 12p+) karyotype concerning 28% of the metaphases at the latter passage (Fig. 3C and Table 1). It is interesting to note that the remaining 34% metaphases harbored the same abnormalities but in different combinations or with less frequent abnormalities. The most observed profile in the I4 cell line was the (39XY, t(Y; 14), +16, 8q+, 5q+) karyotype found in 42% of the metaphases (Fig. 3C) and the (39XY, t(Y; 14), +16, 8q+) found in 32% of the metaphases at the latter passage. Some main rearrangements were conserved between the I3 and I4 lines like the small addition on the long arm of chromosome 8 (8q+) and the chromosome 16 trisomy. In addition to classical cytogenetics, Comparative Genomic Hybridization (CGH) was performed to identify gain or loss of genetic material in I3 and I4 cell lines at early and late passages compared to the initial somatic fibroblasts. Both lines harbored genomic duplications in chromosomes 3, 4 and 10 (Fig. 3D) that we hypothesized to be the source of small additions that were found on chromosomes 8, 9, 5 or 12. Painting of chromosome 8 and 4 revealed indeed that the small addition found on chromosome 8 was constituted by genomic material of chromosome 4 (Fig. 3E). This suggests that the amplification of this specific region, linked to a highly recurrent rearrangement, could give a selective advantage to the carrying cells that became the most represented population. The I20 cell line accumulated different chromosomal rearrangements which finally hit the whole population but the phenomena took place in a more progressive way (Fig. 3A–C). We then performed cytogenetic clonal analysis from early passages of I3, I4 and I20 cell lines. We analyzed the karyotype of three individual clones from each cell line at different times in culture to evaluate the emergence and recurrence of chromosomal abnormalities. After 30 passages, we observed a high percentage of abnormal metaphases in all analyzed clones, but with different abnormalities in each clone, regarding either aneuploidy or translocations (Supplementary File S1). This result suggests that these cell lines are intrinsically unstable but do not seem to be selected for a specific rearrangement or chromosome polyploidy.

### Continuous expression of exogenous factors and poor differentiation of integrative pig iPSLCs

**Figure 4:**
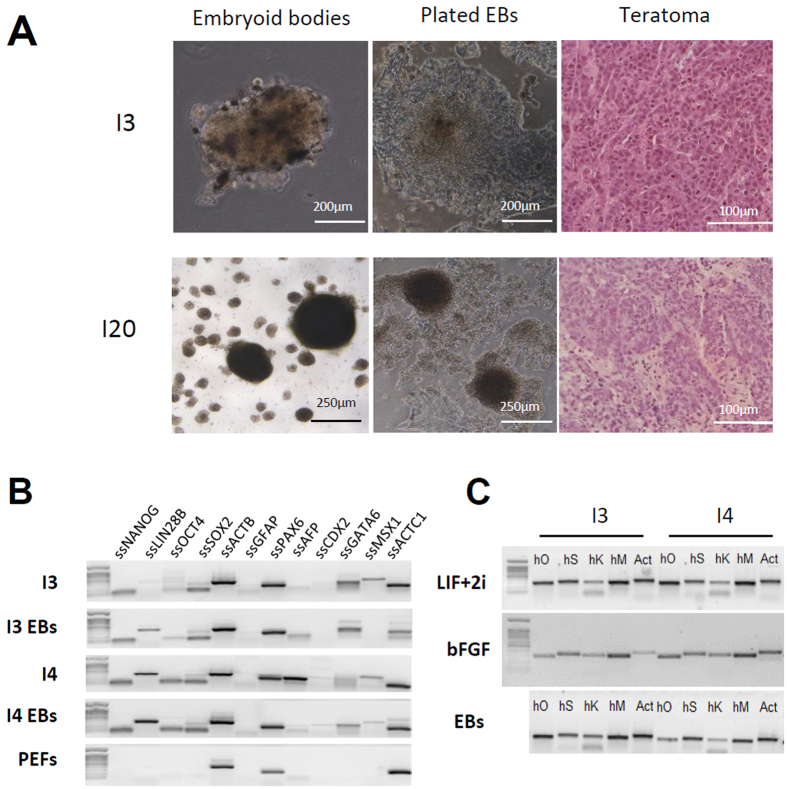
iPS-like cells produced with integrative methods exhibit a poor differentiation potential. (**A**) Embryoid bodies formed by I3 and I20 cell lines poorly differentiate after 5 days of floating culture followed by 10 days in adherent conditions. Teratoma assay leads to the formation of undifferentiated adenocarcinomas. (**B**) Continuous expression of pluripotency genes in the embryoid bodies. (**C**) Continuous expression of exogenous genes in both bFGF and LIF+2i containing media and upon embryoid bodies formation.

To assess the full pluripotent potential of reprogrammed cell lines, we produced embryoid bodies by culturing the cells in suspension under low agitation in a differentiation medium containing neither bFGF nor LIF. None of the three lines derived by retroviral infection were able to produce large and cystic embryoid bodies and the observed structures did not exhibit different cell morphology after moving back to adherent culture conditions (Fig. 4A). Moreover RT-PCR analysis of pluripotency markers and early differentiation markers was found quite similar before and after EB formation for each cell line while they were mainly unexpressed in the embryonic fibroblasts (Fig. 4B). In addition, teratoma formation was unsuccessful as cells injected in immunodeficient mice formed hyperplasic tumors harboring only one cell type (Fig. 4A). One hypothesis for these unsuccessful differentiation assays stays in the continuous expression of reprogramming factors that blocks the cells in a pluripotent-like state. Thus, the 4 exogenous reprogramming factors were found highly expressed in the I3 and I4 lines whatever the culture condition and even after withdrawal of the growth factors sustaining pluripotency and self-renewal during EB formation (Fig. 4C).

### Generation of putative piPSCs by a non-integrative reprogramming technique

**Figure 5:**
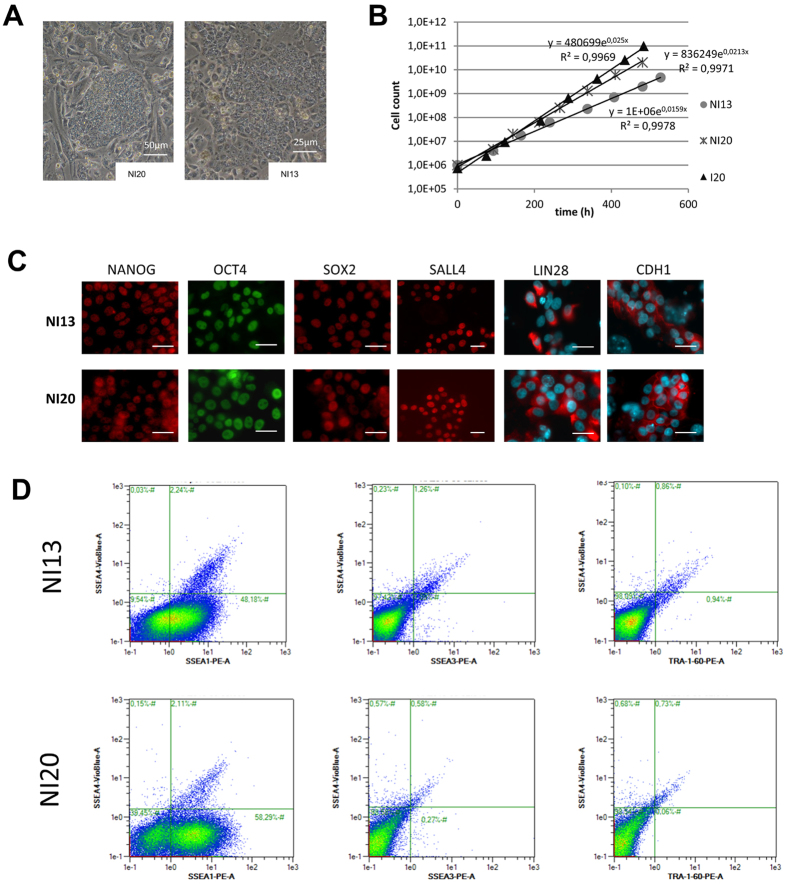
Porcine cell reprogramming using a non-integrative technique. (**A**) iPS-like morphology of NI13 and NI20 lines on feeder cells, scale bar 30 μm. (**B**) NI cell lines grew slightly slower than I20 line. (**C**) Immunofluorescence analysis reveals the nuclear expression of NANOG, OCT4, SOX2 and SALL4 (AF594) and the extra-nuclear expression of LIN28 and CDH1 (AF594 and DAPI nuclei counterstaining) in both NI13 and NI20 lines, scale bar 25 μm. (**D**) Flow cytometry immunofluorescence analysis revealing the strong SSEA1 expression in NI13 and NI20 lines (respectively 50.5% and 60.5%) while SSEA4, TRA160 and SSEA3 are not expressed (less than 3% of the cell population).

**Figure 6:**
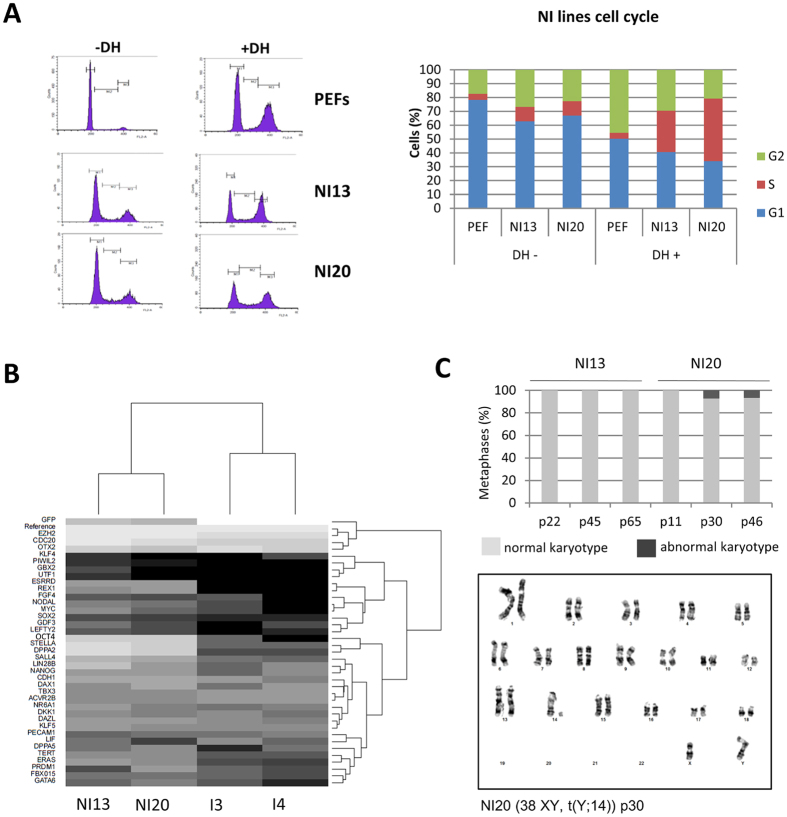
Cell cycle properties, gene expression and karyotype stability of NI-iPSLCs. (**A**) Distribution of cells in the three phases of the cell cycle (M1 = G1 phase; M2 = S phase; M3 = G2/M phase) in PEFs and NI indicating a slight shortening of G1 phase duration in NI lines compared to PEFs, the presence of the G1/S checkpoint in both PEF and NI lines, and the extension of the S phase in NI lines after DSB induction. (**B**) NI lines harbor a stronger expression of the pluripotency-related genes than I lines. Black and white gradations highlight downregulated and upregulated genes, respectively. (**C**) Chromosomal stability of NI lines is not affected by time in culture. Light grey represents the percentage of normal karyotypes and dark grey the percentage of abnormal karyotypes.

To reduce the possible effects of continuous transgene expression on both differentiation potential and chromosomal stability, we next decided to produce porcine iPSCs by using a non-integrative reprogramming technique (NI-iPSCs) based on the use of Sendai virus. Colonies with an iPS-like morphology appeared 5 days after the infection of t(Y; 14) fibroblasts and were picked up between day 15 and day 22 after infection (Fig. 5A). We selected two cell lines, NI13 and NI20, for further characterization. NI13 and NI20 proliferate slower than the control I20 and they necessitate only a 1:6 dilution every 3 days (Fig. 5B). They were alkaline-phosphatase positive and expressed NANOG, OCT4, SOX2, SALL4, LIN28 and CDH1 (Fig. 5C) but SSEA4 could not be detected by immunofluorescence while half of the cell population was SSEA1 positive (Fig. 5D). Their cell cycle was also modified compared to PEFs with a decrease of the G1 phase duration (63% and 67% for NI13 and NI20 lines respectively) and a more balanced repartition of cells in G1 and G2 phases after double-strand breaks induction yet with a surprisingly high amount of cells in the S phase (Fig. 6A). Even though these NI lines were not able to stabilize in LIF+2i culture medium, it is interesting to notice that a huge part of the pluripotency genes tested by quantitative PCR were already more expressed in the NI lines than in the I lines in the piPS medium containing bFGF (Fig. 6B and Supplementary Table S1). The most significant difference in level of expression were observed for *EZH2, DPPA2, STELLA* (*DPPA3*), *CDC20, OCT4* and *SALL4.* Some genes were also expressed only in the NI lines like *ESRRB* and *REX1* (Fig. 6B). Chromosomal stability was increased in the non-integrative lines with all metaphases being (38XY, t(Y; 14)) until late passages in the NI13 line and only 7% of metaphases of the NI20 line harboring aneuploidy starting at passage 30 (Fig. 6C).

**Figure 7:**
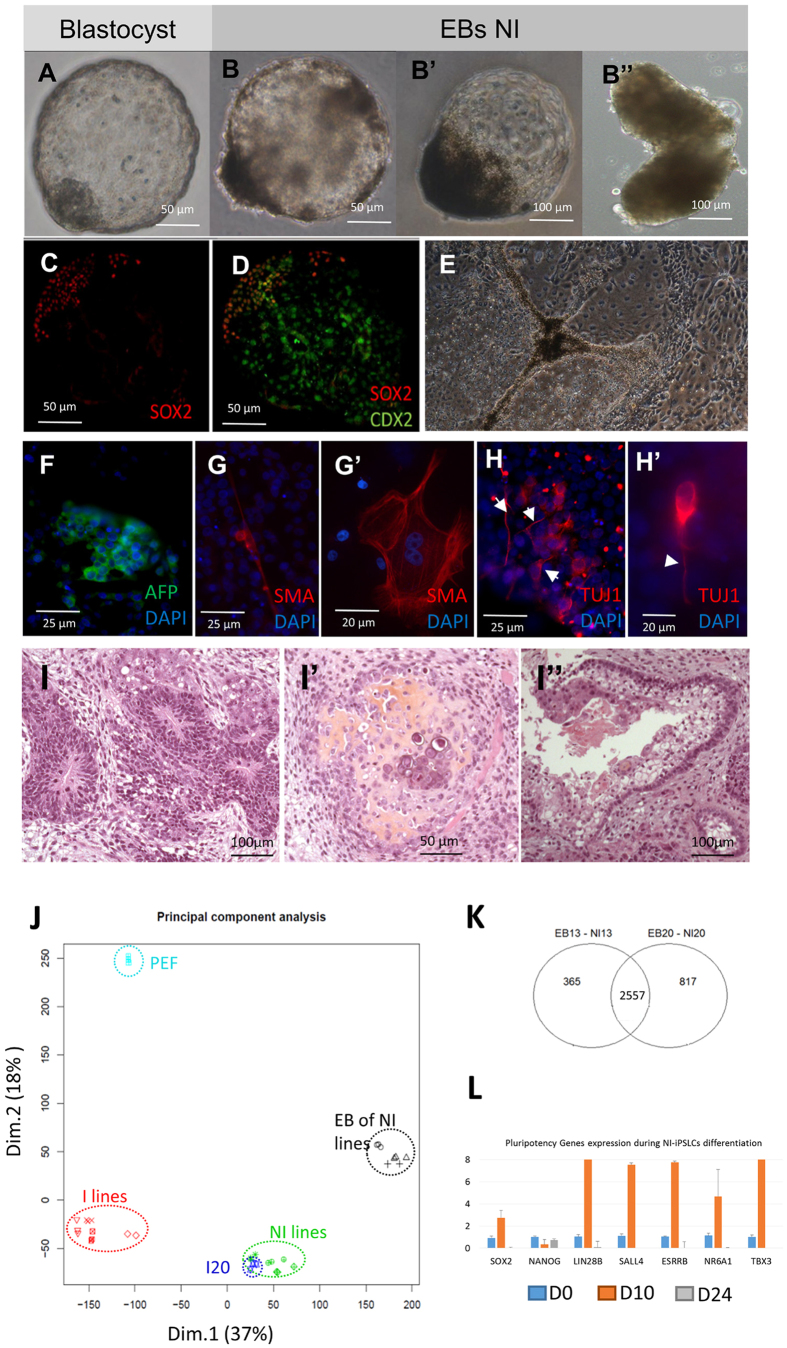
Differentiation potential of NI-iPSLCs. (**A**–**D**) NI-iPSLCs are able to form embryoid bodies with different morphologies (**B**,**B”**) some of them resembling porcine blastocysts (**A**,**B**,**B’**) as they harbor a dense cell mass (like in **B**,**B’**) expressing SOX2 (**C**) and a cellular envelop expressing CDX2 (**D**). (**E**–**H**) In adherent culture conditions, embryoid bodies derived from NI lines differentiate in several cellular types with various morphologies (**E**). Immunostaining for 3-germ layer specific markers highlight the presence of endodermal cells ((**F**) clumps of alpha-fetoprotein (AFP) positive cells in green), of mesodermal cells ((**G**,**G’**) scattered smooth muscle actin (SMA) positive cells in red) or ectodermal cells ((**H**,**H’**) small group of cells with neurons morphology and positive for beta-III tubulin (TUJ1) expression in red, white arrowheads highlight neuron-like projections). (**I**) Histological analysis of teratoma formed by NI lines shows the differentiation into the three germ layers with ectoderm-derived neural crests (**I**), mesoderm-derived chondrocytes with osteoid substance (**I’**) and endoderm-derived epithelium (**I”**). (**J**) Principal Component Analysis of microarray transcriptomic data showing that the repartition of the different expression profiles is explained by the reprogramming technique ((**I**) lines, I20, NI lines) and the level of reprogrammation (reprogrammed, embryoid bodies, non-reprogrammed). (**K**) A large number of differentially expressed probes between NI lines and their respective EB are common for NI13 and NI20 lines. (**L**) Dynamic variation in the expression level of pluripotency-related genes at D0 (blue bars), D10 (orange bars) and D24 (grey bars) of differentiation.

The NI lines appeared to have a higher ability to differentiate. Surprisingly they can form blastocyst-like embryoid bodies that were formed of a dense cellular mass expressing SOX2 and a trophectoderm-like envelope expressing CDX2 (Fig. 7A–D). Once put back in adherent culture conditions these embryoid bodies were able to produce morphologically different type of cells like epithelial cells or adipocyte-like cells containing lipid vesicles (Fig. 7E). Immunostaining for specific markers of the three embryonic germ layers highlight the expanded differentiation potential of NI-iPSLCs. After 15 days of differentiation, we observed cluster of positive cells for the endoderm marker Alpha Fetal Protein (AFP) (Fig. 7F), rare positive cells for the mesoderm marker Smooth Muscle Actin (SMA, Fig. 7G,G’) and frequent TUJ-1 positive neural-like cells (Fig. 7H,H’). Intramuscular injection of NI cells in immunodeficient mice leads to the formation of differentiated teratoma including neural crest cells (Fig. 7I), cartilage (Fig. 7I’) and normal spindle cells epithelia (Fig. 7I”).

We then checked the changes in gene expression during differentiation by performing transcriptomic analysis with a customized 60 K Agilent microarray for several cell lines: PEF, I3 and I4 lines at two different passages (ep = early passage; lp = late passage), I20, NI lines NI12, NI13 and NI20 and their corresponding floating embryoïd bodies. Principal component analysis of the data confirmed the differences in gene expression between the different cell types, with all biological and technical replicates of each cell type clustering together (Fig. 7J). The first axis (37% of the variance) is explained by the variation between I lines, I20, NI lines and embryoid bodies of NI lines, while the second axis (18% of the variance) is explained by the difference between reprogrammed lines (I, NI and I20), embryoïd bodies, and non-reprogrammed cells (PEF). We then performed two-by-two comparison of the probe intensity for several conditions (Supplementary Table S2). It is important to note that several probes can represent the same transcript. Focusing on the differentially expressed probes between NI lines and their respective EBs, we observed that 2557 differential probes were communally differentially expressed for the two NI-iPSLCs cell lines (Fig. 7K). After annotating the probes, we listed 834 common upregulated genes in the embryoïd bodies compared to the iPSCs and 765 downregulated genes. Among upregulated genes were found differentiation markers for endoderm like *AFP, FOXA2, SOX17, HNF3B*, neural ectoderm like *NCAM, CDH2*, non-neural ectoderm like *GATA3, BMP2/3/7*, mesoderm like *HES7, DLL1, PITX2* or *AMY2* (see Supplementary File S2) but also pluripotency-related genes like *ZFP42* (*REX1*), *EPCAM, DPPA5* or *LIFR*. Among downregulated genes were found *LIF, NANOG, DAX1* and *KLF4*. As we previously observed that these cells form first blastocyst-like structure, we suspected a particular kinetic regarding the expression of pluripotency markers. We compared pluripotency gene expression at different differentiation time point (day 0, day 10 in suspension and day 24 in replated EBs). We observed a strong induction of pluripotency genes in the first wave of the differentiation process (Fig. 7L, orange bars) and then a subsequent decrease for most of them (Fig. 7L, grey bars) with few exceptions including *REX1* and *DPPA5*.

**Figure 8:**
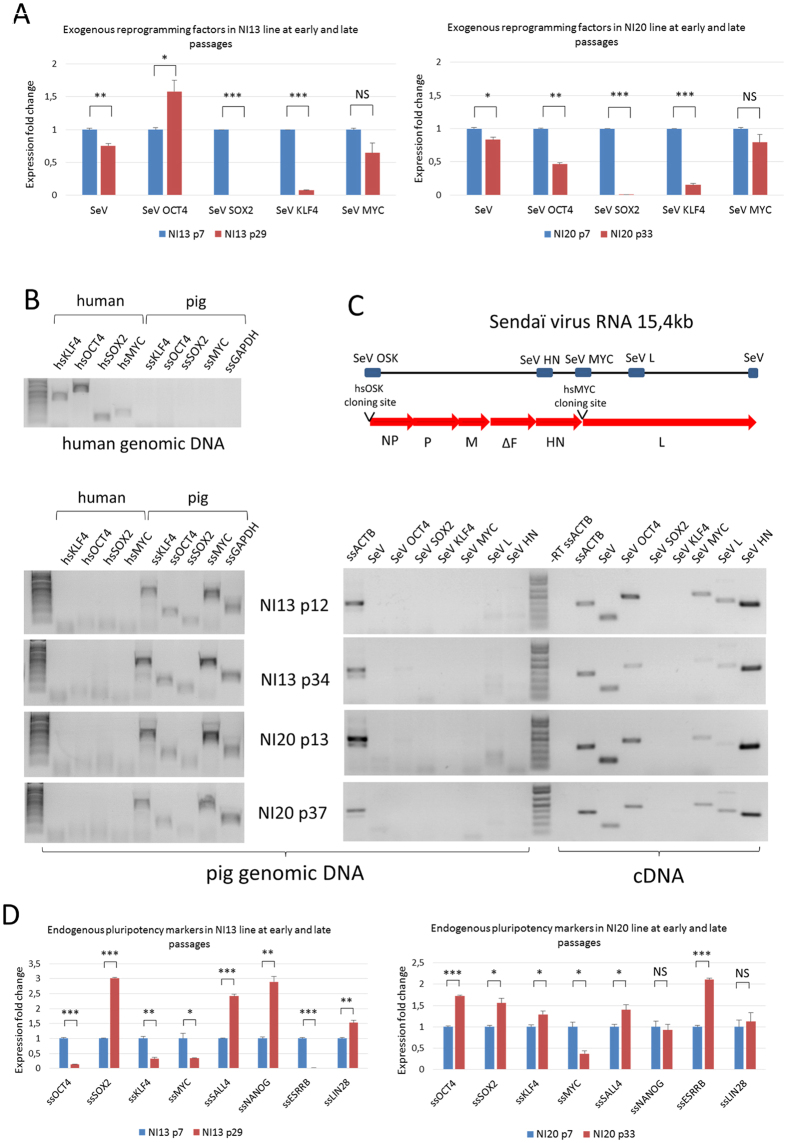
Exogenous reprogramming factors are still expressed but not inserted in genomic DNA of NI13 and NI20 cell lines. (**A**) Real-Time PCR were performed on cDNAs produced from NI13 and NI20 cell lines at early (p7, blue bars) and late (respectively p29 and p33, red bars) passages. Expression of exogenous factors *hsOCT4* (*SeV OCT4*), *hsSOX2* (*SeV SOX2*), *hs KLF4* (*SeV KLF4*), *hsMYC* (*SeV MYC*) and Sendai virus RNA (*SeV*) was quantified between early and late passages in culture. Data are means and SD of two independent experiments (t-test, *P < 0.05; **P < 0.01; ***P < 0.001, NS: not significant). (**B**) PCR analysis was performed on human and pig genomic DNA with primers recognizing specifically the coding sequence of human or pig pluripotency genes. 40 amplification cycles were performed on genomic DNA from human cells or NI13 and NI20 cells at early and late passages. Human sequences were never amplified in the genomic DNA from NI-iPSLCs. (**C**) Representation of the Sendai virus genome and map of the primers used in the PCR analysis. Primers for detecting reprogramming genes are located on one side in the Sendai genome and on the other side in the coding sequence of reprogramming factors, depending on their insertion sites. Three pairs of primers amplifying Sendai virus genes are also mapped. PCRs were performed on genomic DNA from NI13 and NI20 cell lines at early and late passages for 40 amplification cycles. Insertion of exogenous factors *hsOCT4* (*SeV OCT4*), *hsSOX2* (*SeV SOX2*), *hs KLF4* (*SeV KLF4*), *hsMYC* (*SeV MYC*) and Sendai virus genes (*SeV, SeV L and SeV HN*) was never detected. Oppositely, RT-PCRs using RNAs extracted from NI13 and NI20 cell lines at early and late passages clearly show that the whole Sendai virus RNA is expressed and is maintained in the cytoplasm of pig NI-iPSLCs. hsOCT4 and hsMYC expression is maintained among passages, while hsSOX2 and hsKLF4 are not detectable with this assay. (**D**) Real-Time PCR were performed on cDNAs produced from NI13 and NI20 cell lines at early (p7, blue bars) and late (respectively p29 and p33, red bars) passages to quantify relative expression of endogenous pluripotency markers *ssOCT4, ssSOX2, ssKLF4, ssMYC, ssSALL4, ssNANOG, ssESRRB* and *ssLIN28*. Data are means and SD of two independent experiments (t-test, *P < 0.05; **P < 0.01; ***P < 0.001, NS: not significant).

We expected that the use of non-integrative reprogramming technique will facilitate the full extinction of exogenous factors. Thus to evaluate the presence or absence of reprogramming factors in NI-iPSLCs lines, we performed real-time PCR using cDNAs produced from early (p7) and late (p29 and p33 respectively) passages of NI13 and NI20 lines. In both lines, we observed a time-dependent decrease for the Sendai virus (SeV) RNAs coding for *hsKLF4* and the disappearance of SeV RNAs coding for *hsSOX2.* We also observed a slight but not significant decrease of *hsMYC* expression (Fig. 8A). However we still observe a strong expression for *hsOCT4* (Fig. 8 and Supplementary Table S7). Sendai virus (SeV) is a minus-strand RNA virus that can naturally replicate in some cell types like respiratory epithelial cells. Thus, the continuous expression of reprogramming factors may be due either to the persistence of SeV RNA genome in the cytoplasm of NI-iPSLCs or to the insertion of exogenous sequences into the genome of these cells. To test these two hypothesis we performed PCR on genomic DNA from human and from NI13 and NI20 cell lines at early and late passages with primers specifically amplifying either human or pig pluripotency genes. After 40 amplification cycles we were not able to detect any insertion of human reprogramming genes in the genomic DNA of porcine cells while the same primers amplified human reprogramming genes on human genomic DNA (Fig. 8B).

We observed similar results by performing PCR with primers located on one side in the SeV genome and on the other side in the coding sequence of reprogramming factors but also with primers amplifying SeV genes (Fig. 8C). It strongly suggests that neither the exogenous reprogramming genes nor the SeV genome were inserted in the genomic DNA of NI13 or NI20 cell lines. However, we easily amplified exogenous reprogramming factors and SeV genes from reverse transcribed RNAs extracted from NI13 and NI20 cells at early and late passages (Fig. 8A,C). Altogether our data support the hypothesis that the exogenous factors are maintained through the persistence and the replication of the SeV genome in the cytoplasm of NI-iPSLCs rather than genomic insertion.

Then, we evaluate whether the decrease of exogenous genes could affect the expression of endogenous pluripotency players by performing real-time PCR for *ssOCT4, ssSOX2, ssKLF4, ssMYC, ssSALL4, ssNANOG, ssESRRB* and *ssLIN28* between early and late passages in NI13 and NI20 lines. In both cell lines, we observed a strong increase in *ssSOX2* expression and an increase or maintenance of many pluripotency regulators including *ssSALL4, ssNANOG* and *ssLIN28* (Fig. 8). In the NI20 line only *ssMYC* was slightly decreased at p33 compared to p7 while in NI13 line, ss*KLF4* and ss*OCT4* were also decreased. The decrease of exogenous *hsSOX2* and *hsKLF4* between early and late passages was also confirmed in two additional NI-iPSLCs lines (Supplementary Fig. S2) together with the increase or maintenance of most of the analyzed endogenous pluripotency markers (*ssSOX2, ssKLF4, ssSALL4, ssNANOG, ssLIN28*) (Supplementary Fig. S2 and Supplementary Table S7). Together with the maintenance of *hsOCT4* and *hsMYC* among passages we observed a decrease of *ssOCT4* and *ssMYC* in three out of four cell lines suggesting that the continuous expression of these two exogenous factors block the reactivation of their endogenous counterparts. In addition, in order to test whether the exogenous expression of *hsOCT4* can interfere with the differentiation potential of our Ni-iPS-like cells we evaluate the expression levels of exogenous genes using PCR before and after differentiation. We observed a strong decrease of all the exogenous genes in NI13 and NI20 cell lines (Supplementary Fig. S3) contrasting with the constant expression of exogenous genes during differentiation in I3 and I4 lines.

## Discussion

Since the first publications relating the production of pig iPSCs with integrative strategies in 2009^7^,^8^,^9^, laboratories worldwide invested many efforts to produce fully reprogrammed pig pluripotent cells. To date, and to our knowledge, there is no evidence that someone really succeed in such objective. A major issue, yet highlighted in various studies^6^,^13^,^19^, concerns the selection of cells that do not extinct the expression of exogenous genes. This continuous expression of the reprogramming factors strongly interferes with the differentiation potential and the tumorigenicity of the cells.

Thus despite the fact that I3, I4 and I20 cell lines are morphologically similar to ES cells, with a particular cell-cycle, with the expression of core pluripotency genes and the ability to adapt to LIF+2i medium, they fail to differentiate properly and cannot be considered as pluripotent cell lines. Previous reports using integrative techniques succeed in producing iPSLCs able to form teratoma and to differentiate *in vitro* toward the three embryonic layers. In comparison, our iPSLCs produced with integrative techniques are incompletely reprogrammed but allowed us to observe a strong chromosome instability among passages that was not previously described. While I3 and I4 cell lines harbor abnormal karyotypes, we also observed a duplication of a fraction of chromosome 4 that was translocated on q arm of chromosome 8. A similar duplication was previously described in hiPSCs and may provide to the cells a selective advantage^20^. These abnormalities are rarely detected at early passages and have not been detected in the original fibroblast cell line, compromising the hypothesis of a clonal amplification of mosaic abnormalities present in the original somatic cell line^21^. However, through our clonal analysis started at early passage, we observed that rearrangements and/or trisomy were different in each clone and did not reflect a specific selection for determined and recurrent chromosome gain like chromosomes 12 and 17 in human iPSCs^22^,^23^ or chromosomes 8 and 11 in mice^24^ (for a review see Weissbein *et al*.^25^).

We firstly hypothesized that this chromosome instability may be due to an inter-chromosomal effect (ICE) caused by the presence of a constitutive rearrangement, a classical hypothesis explaining chromosome instability in early embryonic cells^26^. But we also observed chromosome instability in the I20 cell line that is derived from a sow with a normal karyotype. In addition this instability was not observed when using a non-integrative strategy even in the presence of this constitutive translocation. Even if controversial results exist in human iPSCs^22^,^27^ and do not clarify whether integrative or non-integrative strategies are causal to genome and chromosome instabilities it seems that it is not the continuous expression of exogenous factors that may explain this instability. Such, in one of these studies, exogenous factors are silenced but the use of integrative reprogramming technologies strongly increases the number of chromosome abnormalities^27^ supporting the fact that viral integrations can cause chromosomal aberrations as shown during papillomaviruses infection^28^. Indeed, the reactivation of endogenous retrovirus and retrotransposable elements presents in the host genome after retro- or lentiviral infection and their genomic integrations^29^ may increase genomic instability and favor the emergence of new rearrangements^30^,^31^.

Even if many groups described the effects of dissociation techniques (Trypsin, Collagenase, Accutase) on the frequency of karyotype abnormalities in human ESC lines^32^,^33^,^34^,^35^ we excluded this hypothesis for various reasons. First manually dissociated human ESCs also exhibit an increasing number of chromosome rearrangements among passages^36^,^37^,^38^. Moreover, we used the same dissociation protocol for all the cell lines and we observed very different outcomes regarding chromosome instability, suggesting a prevailing effect of the reprogramming method and/or efficiency rather than the dissociation methodology for the maintenance of a normal karyotype. Taken together, our results explore for the first time the chromosomal instability of pig iPSLCs at early and late passages and warn against the rapid emergence of chromosome abnormalities upon long-term culture. Although we observed this instability in incompletely reprogrammed cell lines, this point should be taken into consideration and one must be very careful with karyotyping data for long-term cultures.

The use of non-integrative method enabled us to isolate new iPS-like cells that surprisingly express SSEA1 and not SSEA4, similarly to mouse ESCs and recently published pig NI-iPSLCs produced with episomal vectors^13^. It is however difficult to conclude that these cells are equivalent to mouse naïve cells for various reasons. First, early embryonic development of pig conceptus is really different from mouse, rabbit and human models and is associated with specific gene expression patterns in the inner cell mass (ICM) and trophectoderm (TE)^15^,^16^,^39^. Based on expression markers, Hall and Hyttel (2014) 16 proposed that the porcine trophectoderm-covered-epiblast (EPI) may reflect the naïve stem cell state, with strong expression of OCT4, NANOG, CRIPTO and SSEA1 and weak expression of NR0B1 (also known as DAX1) and REX1 (also known as ZFP42), but with very limited expression of genes in classical signaling pathways regulating pluripotency^16^. Transcriptome data from Cao *et al*.^15^ also highlighted differences between pig, human and mouse ICM and TE cells with a specific low SOX2 and NANOG expression in the ICM, low CDX2 expression in the TE, high OCT4 expression in the TE and high GATA6 expression in the morula and ICM cells^15^. Comparative transcriptomic analysis between pig iPS cell lines suggest that previous pig iPS-like cells are closer to the primed than to the naïve state^40^. This study also highlight that i) some pluripotency genes like *TBX3* or *KLF2* are absent from pig iPS-like cells while *TBX3* expression increases chimera contribution and germline competency in mouse iPSCs^41^ and ii) the higher potential for *in vivo* development correlate with EPCAM expression. Our NI cell lines express SSEA1, OCT4, NANOG, GATA6, REX1, NR0B1, TBX3 and EPCAM suggesting that these NI-iPS-like cells could be very close to the porcine naïve state. Thus, these cells can be a useful model to develop and test new culture conditions able to maintain pig pluripotent cells *in vitro*, after downregulation or removal of the remaining hsOCT4 and hsMYC exogenous factors.

The persistent expression of exogenous reprogramming factors despite the use of non-integrative methods was already reported by others including Du and colleagues^13^. After negative selection episomal vector removal, they observed in all their remaining clones the integration of reprogramming factors into the cell genome. Oppositely, in our model, no gene integration was found by PCR-based analysis of genomic DNA in the NI-iPSLCs. Thus, unlike other non-integrative strategies like episomal vectors, transposons or plasmids which are DNA-based vectors, Sendai Virus is a minus-strand RNA virus that can naturally replicate in some cell types^42^. In our study, we used a commercially available system in which the exogenous reprogramming factors are introduced into an F-deficient, temperature sensitive SeV vector which is normally designed to block replication at standard culture temperature leading to passive elimination of the genome through cell passaging and is routinely used for human iPSCs production**43. Our data suggest that, despite the temperature sensitive strain used, the SeV RNA is still replicating in our cells but unlike previous reports, the remaining exogenous reprogramming factors are not inserted in the genomic DNA of pig cells.

Nevertheless, we observed the same dependency on exogenous OCT4 expression to maintain pig cells in a pluripotent state. Altogether it suggests that the reactivation of the endogenous OCT4 is insufficient to autonomously reactivate the endogenous core pluripotency network. This may be due either to remaining epigenetic barriers blocking the reactivation of pig OCT4 as suggested by methylation analysis performed on the pig OCT4 promoter by Du and colleagues^13^ or/and inadequate culture conditions that are not able to maintain pig OCT4 expression during long term culture.

These NI-iPS-like cells also possess a particular differentiation phenotype as they can form in suspension spheroid structures that look like blastocysts. The ability of NI-iPSLCs to form blastocyst-like structure either suggests that two different stem cell populations co-exists in culture or that NI-iPSLCs behave like early embryonic cells with an extended differentiation potential toward embryonic and extra-embryonic fates.

Besides being a model for pig pluripotency, these NI-iPSLCs are the first described to be derived from an infertile animal that carries a constitutive reciprocal translocation. Deriving iPSCs from infertile animals is a promising alternative to study their pathology without having recourse to progeny. Like similar models developed in human^44^,^45^,^46^,^47^, t(Y; 14) NI-iPSLCs open the way for the *in vitro* study of the effects of such translocations on germ cell biology before and during meiosis.

Finally, the use of non-integrative method allows us to isolate new iPS-like cells with better reprogramming: they express higher level of pluripotency markers, exhibit infinite self-renewal without the neo-acquisition of karyotype abnormalities and possess a higher differentiation potential. Moreover we produced for the first time pig iPS-like from an azoospermic boar carrying a reciprocal translocation t(Y; 14). These translocated piPSLCs will then be used as a tool for studying germline commitment and differentiation and the links between chromosomal abnormalities and impaired development at early stages of embryonic development.

## Methods

### Ethics Statement

Our research was conducted in accordance with European Directive 2010/63/EU and experimental protocols (EL-P-MO-MA-09 and EL-P-MO-CH-04) were approved by the Animal Experimentation Ethics Committee for the Poitou-Charentes region (France) (no. CE2012-2).

### Somatic cell culture

Fibroblasts were isolated from adult tissue samples using mechanical dissociation followed by enzymatic digestion with Trypsin 2X. Cells were cultivated in F medium composed of DMEM, 10% Fetal Bovine Serum (FBS), 1% Sodium Pyruvate, 1% Penicillin-Streptomycin, 5 μg/mL Plasmocin Treatment at 37 °C under 5% CO_2_.

### Cell reprogramming and putative piPSCs culture

10^5^ fibroblasts were first infected by the PL-SIN-EOS-C(3+)-EiPlentiviral vector (EOS)^18^,^48^ at a multiplicity of infection of 4 and then cultured in F medium.

On Day 0, 10^5^ EOS+ fibroblasts or non-infected fibroblasts were simultaneously infected by 200000 pV/mL of each of the 4 retroviruses carrying each reprogramming factors -pMX-hOct4, -pMX-hSox4, -pMX-hKlf4, -pMX-hc-Myc-. On Day 2 cells were washed 3 times in PBS. On day 3 cells were passaged with three different concentrations: 5.10^5^, 10^5^ and 5.10^4^ cells per 55 cm^2^ 0.1% gelatin-treated plate containing irradiated STO as feeder cells (13000 cells/cm^2^). Culture medium was then replaced by bFGF medium: DMEM-F12, 10% FBS, 10% Knock Out Serum (KOSR), 1% Sodium Pyruvate, 1% Non-Essentials Amino Acids, 1% Penicillin-Streptomycin, 1% L-Glutamin, 100 μM 2-Mercaptoethanol, 8 ng/mL basic FGF, 5 μG/mL Plasmocin Treatment, and replaced every day. Clones were picked up between D4 and D30 based on their iPS-like morphology and for EOS+ fibroblasts on their GFP expression and puromycin resistance.

For non-integrative cell reprogramming, 10^5^ fibroblasts were transduced by 3.10^6^ CIU of each Sendai virus containing one of the four reprogramming factors (CytoTune^TM^ iPS Reprogramming Kit) in 2 mL of F medium. On day 2 cells were washed and on day 7 cells were diluted in bFGF medium as described before.

Putative porcine iPS cells were cultivated in bFGF medium on 0.1% gelatin treated plate containing irradiated STO feeder cells or in LIF+2i medium on Matrigel and 0.1%-gelatin treated plate. LIF+2i medium is composed as follows: DMEM-F12, 20% KOSR, 1% Sodium Pyruvate, 1% Non-Essentials Amino Acids, 1% Penicillin-Streptomycin, 100 μM 2-mercaptoethanol, 1000 U/mL ESGRO Mouse LIF Medium Supplement, 1% N2 Supplement, 1% B27 Supplement, 1 μM Mek inhibitor PD0325901 (Sigma-Aldrich), 3 μM GSK3β inhibitor 1-azakenpaullone (Sigma-Aldrich), 5 μg/mL Plasmocin Treatment. Cells were passaged by enzymatic dissociation using Accutase.

### Alkaline phosphatase assay

Cells were washed in PBS and fixed with 4% PFA and then incubated 5 minutes at room temperature in NTMT solution (100 mM Tris-HCl pH 9.5, 100 mM NaCl, 50 mM MgCl_2_, 0.1% Triton X-100). Cells were then washed and incubated 20 minutes at room temperature in NTB/BCIP diluted in NTMT (12 μL/mL). Colored cells were finally washed again in KTBT, fixed in 2% PFA and conserved in PBS at 4 °C.

### Embryoid bodies assays

2 million cells were passaged in DIFF medium (DMEM, 10% FBS after decomplementation by heat treatment, 1% Sodium pyruvate, 1% PS, 1% Non-essentials amino acids, 5 μg/mL Plasmocin treatment) in Petri dishes and cultured in suspension under low agitation at 37 °C 5% CO_2_ for 6 days.

### Teratomas formation

3 million cells were injected in immunodeficient mice (NOD.Cg-Prkdcscid Il2rgtm1Wjl/SzJ) by intramuscular injection in the hind leg. Tumors were resected 6 to 12 weeks after injection and fixed in PBS/PFA 4%. After paraffin inclusion, hematoxylin/eosin coloration was performed on tissue cross-section for the histological analysis.

### Immunocytochemistry

Cells were cultured on a coverslip and fixed using 4% PFA. Fixed cells were washed several times in PBS and in PBT (PBS, 0.1% Triton) and blocked in PBT supplemented with 10% FBS. Primary antibodies were diluted in PBT 10% FBS at the following concentrations: anti-NANOG (0.5 μg/mL, Peprotech 500-P236), anti-LIN28 (2 μg/mL, ab63740), anti-OCT4 (2 μg/mL, ab19587), anti-SOX2 (2.5 μg/mL, R&D Systems MAB2018), anti-SSEA4 (4 μg/mL, ab16287), anti-CDH1 (1.25 μg/mL, BD610181), anti-SSEA1 (1/100, Hybridoma Bank MC-480) and revealed with secondary antibodies coupled with Alexa488 and Alexa 561. Nuclei were labeled with Vectashield DAPI mounting medium. Visualization and capture were realized with a Zeiss microscope and the Volocity software. Immunocytochemistry on differentiated embryoid bodies was performed using the 3-Germ Layer Immunocytochemistry kit (Thermo Scientific).

For FACS analysis, 10^6^ cells were suspended in 10 μl of PBS 1% BSA and 1 μl of PE-conjugated or V450-conjugated mouse anti-SSEA1 (clone MC480), rat anti-SSEA3 (clone MC631), mouse anti-SSEA4 (clone MC813) and mouse anti-TRA-1-60, all purchased from BD Biosciences. After 30 minutes incubation at room temperature, cells were washed in PBS and suspended in 200 μl of PBS and analysed by flow cytometry (MACSQuant, Miltenyi). For FACS analysis on differentiating embryoid bodies, dissociated cells were washed in 1xPBS, incubated with DAPI for 2 minutes for live/dead staining, fixed with 1% PFA-PBS for 20 mins at 4 °C and then permeabilized with 0.5% Triton X 100 – PBS for 20 mins at room temperature. Antibody staining was performed in 1% (w/v) BSA-PBS for 20 mins on ice, all antibodies were used in 1:20 dilution. The following antibodies were used: anti - Human/Mouse Brachyury PE (polyclonal goat IgG) R&D Systems and anti-Human Nestin FITC (Monoclonal Mouse IgG1 Clone #196908) R&D Systems. Compensation was performed by using OneComp eBeads (eBioscience).

### Cell cycle and G1/S checkpoint analysis

Cells were suspended in PBS, fixed using 70% cold ethanol and stored at −20 °C for at least 2 h. After centrifugation and resuspension in PBS, cells were treated by 0.1 mg/mL RNAse A for 1 h at 37 °C. DNA was next stained by 25 μg/mL Propidium iodide. For checkpoint analysis, cells were previously treated for 24 h by 65 ng/mL doxorubicin hydrochloride (DH) which induces DNA breaks. Ratio of cells in each cell cycle phase was measured by flow cytometry (FACScalibur, BD Bioscience).

### Gene expression analysis

Total RNA from cell pellets was extracted using the Nucleospin RNA kit (Macherey-Nagel). Quantity and quality of RNA were evaluated by NanoDrop dosage and gel electrophoresis. RNA was reverse transcribed and conventional PCR was done with primers and PCR parameters described in Supplementary Tables S3 and S4 49,50. Primers for Real-time PCR were designed by the Primer3 online software (Supplementary Table S5). After mixing 2 μL of cDNA, primers and SYBR Green, high-throughput Real-Time PCR was realized on the BioMark HD System (Fluidigm). Results were visualized on the Real-Time PCR Analysis software (Fluidigm) and analyzed by the 2ΔΔCt method using the geometrical mean of Ct values for the three reference genes (ACTB, GAPDH and TBP) as a reference and the I3 cell line in LIF+2i medium as a calibrator. Statistical analysis was performed by *t*-test for two-by-two comparisons and 2-way ANOVA for multiple comparisons. Conventional real-time PCR was performed on an Agilent Mx3005p system. Results were visualized on the MxPro software (Agilent) and analyzed by the 2ΔΔCt method using the geometrical mean of Ct values for the reference genes RPL4 and HPRT as a reference^49^.

### PCR on Genomic DNA

Genomic DNA from the different cell lines was purified using the DNeasy Blood and Tissue Kit (Qiagen). Control human genomic DNA was purchased from Roche. PCR was performed using Taq Polymerase (Thermo Scientific) with primers designed to specifically amplify human or porcine genes and described on Supplementary Table S4. Amplification was performed over 40 cycles and annealing temperature was defined according to primers melting temperatures.

### Microarray analysis

Total RNA from cell pellets was extracted in triplicate using the Nucleospin RNA kit (Macherey-Nagel). Quantity and quality of RNA was evaluated by NanoDrop dosage and Bioanalyser analysis (Agilent Technologies). Samples were then marked by Cyanine3 fluorochrome and converted in cDNA before hybridization on the 60 K customized porcine microarray from Agilent^51^. Data were then filtered to keep only spots of sufficient intensity and quality for at least 2 out of 3 technical replicates. Intensity values were log-transformed and normalized by the quantile normalization method. Differential analysis was then performed using the Limma package (Bioconductor). Threshold parameters were defined as follow: maximal adjusted p-value (Benjamini& Hochberg) of 0.01 and minimal log-fold change (lfc) of 2.

### Karyotype analysis

Karyotype analysis were performed like described in Osteil *et al*.^52^. Briefly, at 80% confluence approximately, cells were incubated with 0.4 μg/mL Colcemide for 3 hours at 37 °C and detached. Cells were resuspended in KCl 0.075 M for an hypotonic shock at 37 °C for 2 minutes and pre-fixed by adding a few drops of fresh fixing solution (Ethanol:Acetic acid; 3:2). Cells were then dropped on cold microscope slides for metaphase spreading. G-banding of the metaphase were obtained by successive treatments of the slide: 1′15′′ wash in 0.025% trypsine at room temperature, quick wash in PBS, and a final 8 to 9 minutes incubation in Giemsa solution. Pictures of metaphases were captured by the Cytovision system and the karyotyping analysis was performed using the Genus software.

### Comparative genomic hybridization

500 ng of genomic DNA from two cell lines were were labelled by random priming by direct labeling with fluorescent dUTP (Alexa 488 for one cell line and Alexa 568 for the other). Control metaphases from porcine lymphocytes spread on microscope slide were denatured 5 minutes at 73 °C in 2× SSC 70% Formamide. Fluorescent DNA probes were denaturated in boiling water for 7 minutes and incubated 45 minutes at 37 °C. Next, DNA probes were hybridized on metaphases for 3 day at 37 °C. Slides were finally washed several times in 2× SSC 50% Formamide at 45 °C and mounted. Metaphases were then captured and karyotyped in reverse-DAPI using Genus (Cytovison) to calculate the ratio of green and red fluorescence along the chromosomes.

### Fluorescence *in situ* hybridization

Chromosomal painting probes (2 μg)^53^ were labelled by random priming by direct labeling with fluorescent dUTP. Metaphases slides were treated as follows: 30 minute wash in 2× SSC, 1 h incubation in diluted RNAse A in 2× SSC, 9 minutes incubation in diluted proteinase K in 2× SSC (0.1 μg/mL), two washes in 2× SSC, dehydration by successive 3 minutes washes in ethanol (70%, 80%, 90%) and cold drying. Probes and metaphases were then hybridized in a Hybridizer (Dako) programmed in two steps: 5 minutes of denaturation at 75 °C and 24 to 48 h of hybridization at 37 °C. Slides were next washed two times 15 minutes in 2× SSC at room temperature, three times 3 minutes in 2× SSC at 45 °C. Finally, mounting medium with DAPI was dropped between slide and coverslip for further microscopic observations.

### Statistical analysis

Error bars in figures represent standard deviation. Differences in gene expression between two conditions from RT-qPCR data were examined using Student’s paired *t*-test and a p-value < 0.05 was considered significant. Microarrays results were analysed using the R Bioconductor package based on linear models for microarray data (limma): differences between two groups were considered significant when the p-value (moderated t-statistic) was inferior to 0.01 after Benjamini&Hochberg correction for multiple testing.

## Additional Information

**Accession Codes**: Microarrays GEO Accession codes: GSE71792.

**How to cite this article**: Congras, A. *et al*. Non integrative strategy decreases chromosome instability and improves endogenous pluripotency genes reactivation in porcine induced pluripotent-like stem cells. *Sci. Rep.* **6**, 27059; doi: 10.1038/srep27059 (2016).

## Supplementary Material

Supplementary Information

Supplementary Information

## Figures and Tables

**Table 1:** Main karyotypes frequencies in the different cell lines.

	I3 p9	I3 p110	I4 p9	I4 p111	NI13 p22	NI13 p65	NI20 p11	NI20 p46	I20 p15	I20 p73
2n = 38, XY, T(Y/14)	82%	0%	72%	0%	100%	100%	100%	93%		
2n = 39, XY, T(Y/14), +16	6%	0%	4%	0%	0%	0%	0%	7%		
2n = 37, XY, T(Y/14), der14−, 8q+	0%	38%	0%	0%	0%	0%	0%	0%		
2n = 39 = , XY, T(Y/14), 9q+, 12p+, +16	0%	28%	0%	0%	0%	0%	0%	0%		
2n = 39, XY, T(Y/14), 5q+, 8q+, +16	0%	0%	0%	42%	0%	0%	0%	0%		
2n = 39, XY, T(Y/14), 8q+, +16	0%	0%	0%	32%	0%	0%	0%	0%		
2n = 38, XX									83%	0%
2n = 39, XX, +10, 6q−, 7q+, 9q−									0%	40%
2n = 39, XX, +10 6q−, 9q−									0%	47%
