# Corrigendum: Non integrative strategy decreases chromosome instability and improves endogenous pluripotency genes reactivation in porcine induced pluripotent-like stem cells

**DOI:** 10.1038/srep46931

**Published:** 2018-01-08

**Authors:** Annabelle Congras, Harmonie Barasc, Kamila Canale-Tabet, Florence Plisson-Petit, Chantal Delcros, Olivier Feraud, Noufissa Oudrhiri, Eva Hadadi, Franck Griscelli, Annelise Bennaceur-Griscelli, Ali Turhan, Marielle Afanassieff, Stéphane Ferchaud, Alain Pinton, Martine Yerle-Bouissou, Hervé Acloque

Scientific Reports 6: Article number: 2705910.1038/srep27059; published online: 06 01 2016; updated: 01 08 2018

In the original version of this Article, the Supplementary Information file containing the supplementary figures and tables was omitted. This has been corrected in the HTML version of the Article; the PDF version was correct at the time of publication.

